# The Effects of Eccentric and Plyometric Training Programs and Their Combination on Stability and the Functional Performance in the Post-ACL-Surgical Rehabilitation Period of Elite Female Athletes

**DOI:** 10.3389/fphys.2021.688385

**Published:** 2021-07-02

**Authors:** Sofien Kasmi, Hassane Zouhal, Raouf Hammami, Cain C. T. Clark, Anthony C. Hackney, Amri Hammami, Mokhtar Chtara, Sabri Gaied Chortane, Fatma Zohra Ben Salah, Urs Granacher, Omar Ben Ounis

**Affiliations:** ^1^Tunisian Research Laboratory “Sport Performance Optimization”, National Center of Medicine and Science in Sports, Tunis, Tunisia; ^2^Department of Physiotherapy, Posturology and Functional Rehabilitation, National Center of Medicine and Science in Sports, Tunis, Tunisia; ^3^M2S Laboratory (Movement Sport Science Laboratory), Université Rennes, Rennes, France; ^4^Higher Institute of Sport and Physical Education of Ksar Saïd, Tunis, Tunisia; ^5^Research Laboratory: Education, Motor Skills, Sports and Health (EM2S, UR15JS01), Higher Institute of Sport and Physical Education of Sfax, University of Sfax, Sfax, Tunisia; ^6^Centre for Intelligent Healthcare, Coventry University, Coventry, United Kingdom; ^7^Department of Exercise and Sport Science, The University of North Carolina at Chapel Hill, Chapel Hill, NC, United States; ^8^Laboratory of Physiology, Ibn El Jazzar Medical Faculty of Sousse, Sousse, Tunisia; ^9^Department of Physical Medicine and Rehabilitation, Institute of Orthopedics “M.T. Kassab,” Manouba, Tunisia; ^10^Division of Training and Movement Sciences, University of Potsdam, Potsdam, Germany

**Keywords:** return-to-sport, anterior cruciate ligament, eccentric-plyometric, training, stability, functional performance

## Abstract

**Background:**

The standard method to treat physically active patients with anterior cruciate ligament (ACL) rupture is ligament reconstruction surgery. The rehabilitation training program is very important to improve functional performance in recreational athletes following ACL reconstruction.

**Objectives:**

The aims of this study were to compare the effects of three different training programs, eccentric training (ECC), plyometric training (PLYO), or combined eccentric and plyometric training (COMB), on dynamic balance (Y-BAL), the Lysholm Knee Scale (LKS), the return to sport index (RSI), and the leg symmetry index (LSI) for the single leg hop test for distance in elite female athletes after ACL surgery.

**Materials and Methods:**

Fourteen weeks after rehabilitation from surgery, 40 elite female athletes (20.3 ± 3.2 years), who had undergone an ACL reconstruction, participated in a short-term (6 weeks; two times a week) training study. All participants received the same rehabilitation protocol prior to the training study. Athletes were randomly assigned to three experimental groups, ECC (*n* = 10), PLYO (*n* = 10), and COMB (*n* = 10), and to a control group (CON: *n* = 10). Testing was conducted before and after the 6-week training programs and included the Y-BAL, LKS, and RSI. LSI was assessed after the 6-week training programs only.

**Results:**

Adherence rate was 100% across all groups and no training or test-related injuries were reported. No significant between-group baseline differences (pre-6-week training) were observed for any of the parameters. Significant group-by-time interactions were found for Y-BAL (*p* < 0.001, ES = 1.73), LKS (*p* < 0.001, ES = 0.76), and RSI (*p* < 0.001, ES = 1.39). Contrast analysis demonstrated that COMB yielded significantly greater improvements in Y-BAL, LKS, and RSI (all *p* < 0.001), in addition to significantly better performances in LSI (all *p* < 0.001), than CON, PLYO, and ECC, respectively.

**Conclusion:**

In conclusion, combined (eccentric/plyometric) training seems to represent the most effective training method as it exerts positive effects on both stability and functional performance in the post-ACL-surgical rehabilitation period of elite female athletes.

## Introduction

Anterior cruciate ligament (ACL) injury incidence and patterns have been widely studied across many sports in women. Despite the fact that male athletes are less vulnerable to this type of injury, the associated risk factors are the same as they are in females with low knee flexion angles, greater knee valgus, and high ground reaction forces upon landing (Lin et al., 2012). As such, rates of non-contact ACL tears range from two to four times greater in female basketball and soccer athletes than in males playing the same sports (Ireland, 1999). Despite these risks, evidence suggest that a multicomponent training program can offset the risk of sustaining ACL injuries in either sex (Padua et al., 2018).

In this context, recent advances in rehabilitation following ACL surgery continue to progress, with current emphasis being on patients achieving immediate weight-bearing (Ardern et al., 2011), range of motion (Wilk et al., 1992), progressive muscular strengthening (Chmielewski et al., 2016), and dynamic stability and neuromuscular control through exercise (Wilk et al., 2012). Subsequent to surgery, patients engage in extensive rehabilitation programs with a view to enhancing the strength, stability, flexibility, and muscular power of the injured limb (Cervenka et al., 2018). Typically, a 4-to-9-month rehabilitation program is implemented after this surgery (Kinikli et al., 2014). Female athletes are more vulnerable to sustaining ACL injuries than their male counterparts, seemingly because of anatomical, hormonal, and neuromuscular differences (Hewett, 2000). Of these factors, only the neuromuscular component can be modified through preventive exercise with neuromuscular training, which includes plyometrics and strength work, being effective at preventing ACL injury in female athletes (Yoo et al., 2010). Accordingly, further study using more advanced exercise such as eccentric, concentric, and combined modalities is required to develop a relevant training protocol of appropriate content and intensity.

A standard rehabilitation program typically involves concentric and eccentric exercises, with a recent critical review, indicating that eccentric training, such as Nordic hamstring curls, may be most effective in restoring hamstring strength (Gokeler et al., 2013). Indeed, in patients (*n* = 33) with autogenous hamstring ACL reconstructions, Kinikli et al. (2014) demonstrated greater improvements in measures of vertical jump (*p* = 0.012), single-leg hop (*p* = 0.027), and Lysholm Knee Scale (LKS) (*p* = 0.002) after 12 weeks of progressive eccentric and concentric training.

Furthermore, these authors showed that neuromuscular training can be combined with strength training to maximize outcomes (Gokeler et al., 2013); however, muscle atrophy of the lower limb is observed in patients after ACL reconstruction and can persist up to 1 year after surgery and completion of a 6-month guided rehabilitation protocol (Aurora et al., 2014). Physical rehabilitation programs tend to start immediately after surgery and last from 12 to 24 weeks, supporting the regeneration of musculotendinous tissue at harvest sites (Lee et al., 2016).

However, it is usually only 3–4 months after reconstruction that the focus of such rehabilitation programs shifts to the enhancement of muscular volume and strengthening (Lindstrom et al., 2013). Further to this, muscle morphology (Friedmann-Bette et al., 2018), balance and stability (Cervenka et al., 2018), and psychosocial status (Kinikli et al., 2014; Chmielewski et al., 2016) are considered to be the components tested most often during rehabilitation. Despite this, little is known about morphological changes, QOL, and functional performance alterations in the quadriceps femoris muscle after different combined rehabilitation programs. Friedmann-Bette et al. (2018) conducted a 12-week standardized rehabilitation program following ACL reconstruction in recreational athletes (*N* = 37). After the training period, the researchers deployed a twice-weekly program of either conventional eccentric or eccentric overload resistance training with additional load, using supervised one-legged leg-press training. The authors found that eccentric overload resistance training led to significantly greater muscle hypertrophy than conventional eccentric resistance training. However, the training program induced at the same time a slower muscle phenotype for strong and fast movements. With this in mind, further studies are required that use more advanced exercise programs such as eccentric, concentric, and combined modalities to develop a relevant training protocol of appropriate content and intensity.

Thus, the aim of this study was to assess the effects of 6 weeks of eccentric training, plyometric training, or a combination of these two modalities, on measures of dynamic stability (Y-BAL), psychological readiness to return to sport (RTS) [LKS and return to sport index (RSI)], and leg symmetry index (LSI) for the single leg hop test in the post-ACL-surgical rehabilitation period of elite female athletes. Based on a relevant literature data (Lindstrom et al., 2013; Kinikli et al., 2014; Chmielewski et al., 2016; Friedmann-Bette et al., 2018), we hypothesized that the combined (eccentric/plyometric) training would lead to greater improvements in the outcome measures of interest in elite female athletes after rehabilitation ACL surgery.

## Materials and Methods

### Participants

Forty-eight elite female athletes who had undergone an ACL reconstruction participated in this study (Table 1). Only 40 athletes completed the training program (eight athletes were eliminated from study due to their absenteeism). Of the trained athletes, 38 underwent surgery on their left limb, and the remaining two had surgery on their right. All participants were matched for age, gender, body mass index (BMI), and training experience. Female athletes performed a systematic sport practice at international level and were members of the Tunisian national team in their respective sport.

**TABLE 1:** Anthropometric characteristics of the study participants.

	**Age (years)**	**Height (cm)**	**Mass (kg)**	**BMI (%)**
**CON (*n* = 10)**	20.3 ± 3.3	171.6 ± 9.6	64.2 ± 7.0	18.6 ± 1.6
**ECC (*n* = 10)**	20.3 ± 3.1	170.7 ± 8.0	67.1 ± 8.0	19.6 ± 1.5
**PLYO (*n* = 10)**	20.3 ± 3.4	172.1 ± 7.7	65.5 ± 9.1	18.9 ± 2.0
**COMB (*n* = 10)**	20.2 ± 3.7	172.3 ± 9.6	65.4 ± 11.1	18.8 ± 2.3

*Values are means and standard deviations (SD); traditional rehabilitation training = CON; eccentric-only training = ECC; plyometric-only training = PLYO; combination of eccentric and plyometric training = COMB; BMI = body mass index.*

Participants’ inclusion criteria were as follows:

1. female athletes who performed systematic sport practice at an international level and were members of the Tunisian national team in their respective sport (e.g., athletics, judo, team sports, etc.);
2. on average, athletes exercised between six and eight times per week including competition; and
3. athletes with non-contact ACL injury during sporting activity (training and/or competition).

Participants’ exclusion criteria were as follows:

1. recreational athlete or untrained individual;
2. athletes with less than five training sessions per week;
3. ACL injury caused by contact;
4. athletes who were operated with techniques other than the bone patellar-tendon, bone graft (BPTB), or who were operated by different surgeons and rehabilitated by several physical therapists;
5. athletes with a history of muscle or joint injuries;
6. athletes with poor attendance rates (below 80%);
7. athletes who had already followed a pre-operative rehabilitation program;
8. athletes with complication after surgery;
9. pregnant athletes and those who were taking anti-inflammatory drugs or muscle relaxers were also excluded.

Participants provided informed consent after thorough explanation of the objectives and scope of this project, the procedures, risks, and benefits of the study.

The study was conducted according to the Declaration of Helsinki, and the protocol was fully approved by the Ethics Committee of the National Centre of Medicine and Science of Sports of Tunis (CNMSS-LR09SEP01) before the commencement of the assessments.

An *a priori* power analysis (*N.B.*, expected SD of residuals = 20 AU for LKS, desired power = 0.90, and alpha error = 0.01), using GPower 3.1 software (Version 3.1, University of Dusseldorf, Germany) (Faul et al., 2009), was computed to simulate a statistically significant group-by-time interaction for our primary outcome LKS (Kinikli et al., 2014). The analysis revealed that a total sample size of *N* = 36 would be sufficient to achieve a medium-sized group-by-time interaction effects. Accordingly, 40 participants were enrolled to account for potential dropouts due to individual withdrawing from the study.

### ACL Surgery and Rehabilitation

All surgeries were performed at the Hospital Ortopédico (Alyssa, Tunisia) by the same two knee surgeons who had at least 20 years of technical experience with ACL reconstruction. Two weeks after surgery and 12 weeks before the training program, all participants received the same rehabilitation protocol (phases 1, 2, 3, and 4) consisting of edema and inflammation control, expansion of range of motion, improvement of stability, and training of muscle strength (Shelbourne et al., 1991).

### Randomization

Participants were randomly allocated to three experimental groups and a control. Group allocation was realized by adjusting for sex, age, and BMI of the study sample. In addition, the order of each trial was changed randomly between participants, in order to avoid learning effects and fatigue. After 14 weeks of rehabilitation post ACL surgery, this latter procedure was performed by an independent researcher who was not involved in the patients’ evaluation or rehabilitation.

### Experimental Design

**FIGURE 1:**
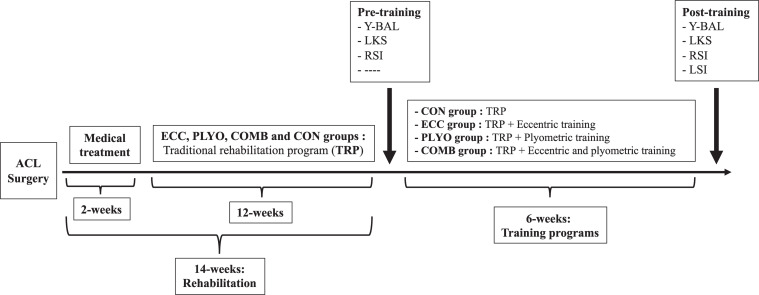
Experimental protocol adopted in the present study. Y-BAL, dynamic stability; LKS, Lysholm Knee Scale; RSI, return to sport index (RSI); LSI, leg symmetry index for the single leg hop test for distance.

Two weeks after ACL surgery, female athletes who had completed 12 weeks of rehabilitation participated in the 6-week training study (Figure 1). The three experimental groups were as follows: (1) the eccentric group (ECC, *n* = 10), (2) the plyometric group (PLYO, *n* = 10), and (3) the combined eccentric and plyometric group (COMB, *n* = 10).

The control group (CON, *n* = 10) was recruited from the same elite female athletes who had undergone an ACL reconstruction and was also tested and was instructed to follow their traditional program (Shelbourne et al., 1991). Testing was conducted before and after the 6-week training programs and included the assessment of Y-BAL, LKS, and RSI. However, LSI was assessed just after the 6-week training programs. For the applied tests, two trials were performed with approximately 2 min rest in between trials. The best trial result was used in the analysis.

### Training Programs

The ECC, PLYO, and the COMB groups were required to participate in two additional weekly training sessions (i.e., ∼60 min per session) over a period of 6 weeks (12 training sessions in total) (Table 2) in addition to the traditional program (Shelbourne et al., 1991). The training volume (training weeks, sets, repetitions, and duration) was equal between the experimental groups. The training instruction carried out on patients was developed from a protocol reported in the literature (Lee et al., 2016; Wilk and Arrigo, 2017) that was adapted from the work of Shelbourne and Nitz (1990). Each training session began with a standardized 15-min warmup, including submaximal intensity running, dynamic stretching, calisthenics, and preparatory exercises (e.g., balance and landing, squatting, and jumping exercises) at a progressively increased intensity. All the participants were under care at the National Medical Sports Center of Tunisia, using the same periodization scheme and the same physical therapist. It was carried out under the supervision of a certified physical therapist in the National Medical Sports Center of Tunisia.

**TABLE 2:** Plyometric, eccentric, and combined (eccentric/plyometric) training programs.

	**Week 1**	**Week 2**	**Week 3**	**Week 4**	**Week 5**	**Week 6**
**Plyometric program**						
Standing vertical hops	2S × 8R	2S × 10R	3S × 8R	2S × 8R		
*Countermovement jump* (CMJ)	2S × 8R	2S × 10R	3S × 8R	2S × 8R		
Depth jumps	2S × 8R	2S × 10R	3S × 8R	2S × 8R		
Multiple two-foot hurdle jumps (hurdle height 0.55 m)	2S × 8R	2S × 10R	3S × 8R	2S × 8R	3S × 8R	3S × 10R
Two-foot jumps forward and backward					3S × 8R	3S × 10R
Single foot jumps					3S × 8R	3S × 10R
Lateral two-foot jumps					3S × 8R	3S × 10R
Total volume	64	80	96	64	96	120
**Eccentric program**						
Nordic hamstring exercise	2S × 8R	2S × 10R	3S × 8R	2S × 8R	3S × 8R	3S × 10R
Eccentric hamstring curl	2S × 8R	2S × 10R	3S × 8R	2S × 8R	3S × 8R	3S × 10R
Quadriceps eccentric leg Extension	2S × 8R	2S × 10R	3S × 8R	2S × 8R	3S × 8R	3S × 10R
Glute-hamstring raise	2S × 8R	2S × 10R	3S × 8R	2S × 8R	3S × 8R	3S × 10R
Total volume	64	80	96	64	96	120
**Combined program**						
-Standing vertical hops	1S × 6R	1S × 8R	1S × 8R	1S × 6R	1S × 8R	2S × 6R
-Nordic hamstring exercise 7	1S × 10R	2S × 6R	2S × 8R	1S × 10R	2S × 8R	3S × 6R
-Depth jumps	1S × 6R	1S × 8R	1S × 8R	1S × 6R	1S × 8R	2S × 6R
-Quadriceps eccentric leg tension	1S × 10R	2S × 6R	2S × 8R	1S × 10R	2S × 8R	3S × 6R
-Single foot jumps	1S × 6R	1S × 8R	1S × 8R	1S × 6R	1S × 8R	2S × 6R
-Eccentric hamstring curl	1S × 10R	2S × 6R	2S × 8R	1S × 10R	2S × 8R	3S × 6R
-Lateral two-foot jumps	1S × 6R	1S × 8R	1S × 8R	1S × 6R	1S × 8R	2S × 6R
-Glute-hamstring raise	1S × 10R	2S × 6R	2S × 8R	1S × 10R	2S × 8R	3S × 6R
Total volume	64	80	96	64	96	120

*S = sets; R = reps.*

### Dynamic Stability

**FIGURE 2:**
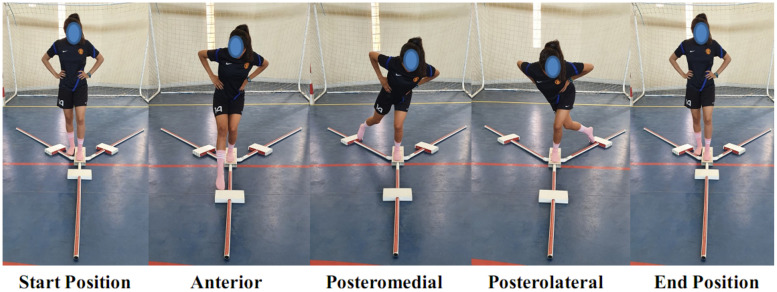
The Y-balance test.

The Y balance test is a dynamic balance (Y-BAL) test that requires subjects to maintain single leg stance while reaching as far as possible with the contralateral leg in three different movement directions (i.e., anterior, posteromedial, and posterolateral) (Figure 2).

For this purpose, participants stood on the dominant leg, with the most distal aspect of their great toe on the center of the grid and began in a unilateral stance with their big toe behind the line on the center of the tape. Distances were then recorded by pushing a moveable floor target with the big toe in the three different directions, with trials performed with the dominant leg. Participants were required to keep the heel of the non-reaching leg on the platform, maintaining the balance in a single leg stance, and returning the reaching foot back to the starting point before attempting the next direction. Maximal reaching distances were recorded to the nearest 0.5-cm marker on the Y-balance test kit. Balance performance was expressed as the Y-balance composite test score (YBT-CS), obtained by dividing the sum of the maximal reached distances in each of the three directions, by three times the length of the lower limb (Fusco et al., 2020). Following the completion of the test trials, each participant was given a 1-min rest period and then conducted two additional test trials in each direction.

### Lysholm Knee Scale

The LKS is an eight-item questionnaire designed to evaluate patients after knee ligament injury. It is scored on a 100-point scale from 0 to 100 (worst to best symptoms, respectively), with 25 points attributed to pain, 15 to locking, 10 to swelling, 25 to instability, 10 to stair climbing, and 5 points each to limping, use of a support, and squatting (Lysholm and Gillquist, 1982; Tegner and Lysholm, 1985).

### Return to Sport Index (RSI)

The RSI questionnaire considered three elements that have previously been correlated with returning to sport: emotions, confidence in one’s performance, and evaluation of risk. It is composed of 12 questions anchored to a visual 10-point Likert scale, in the form of boxes to be ticked, ranging from 0 to 10. The total score was obtained by adding the values of the 12 responses, then obtaining their relationship to 100 to obtain a percentage, with high scores corresponding to a positive psychological response. The tool possesses a high degree of internal consistency (Chronbach’s α = 0.96) (Vealey et al., 1998).

### LSI for the Single Leg Hop Tests

Data from unilateral lower limb hop tests were collected and performed as described by Noyes et al. (1991), including one jump with and without hands, triple jump, triple cross jumps for distance, and 6 m for time (Gustavsson et al., 2006).

For the single one leg hop test for distance, the aim is to jump as far as possible on a single leg, without losing balance and landing firmly. The participant stands on the leg to be tested, jumps horizontally at maximal effort, and lands on the same limb, without touching the ground with hands and without tripping. The distance jumped was measured at the level of the big toe and recorded to the nearest centimeter from a standard tape measure that was permanently attached to the floor.

With the triple jump test, the patient stood on one leg, performed three maximal and consecutive horizontal jumps, and landed on the same foot with the same procedure as performed during the single jump. The total covered distance was measured and recorded.

In the triple hop test, the aim is to jump as far as possible on a single leg three consecutive times, without losing balance and landing firmly. The distance was measured from the start line to the heel of the landing leg. The goal is to have a less than 10% difference in hop distance between the injured limb and uninjured limb (Noyes et al., 1991). For the 6-m hop for time, this is a timed test performed over a distance of 6 m. Each subject was invited to use linear, large, and forceful one-legged hopping motions, to propel their body toward the measured distance as quickly as possible. The total covered distance was measured. The mean values and the LSI were calculated as previously described for the single jump test. The LSI was calculated as the mean score for the operated limb/non-operated limb × 100% (Gustavsson et al., 2006; Myers et al., 2014). This score indicates cessation of rehabilitation and RTS (Thomee et al., 2011; Logerstedt et al., 2014). All jump tests were performed until two successful hops were obtained for each leg, with the starting order of the right or the left leg randomly assigned to the participants. Each participant was given two practice trials before the test started, with 1-min rest between trials (Noyes et al., 1991; Gustavsson et al., 2006).

### Statistical Analyses

Data are presented as means and standard deviations (SD). Data normality was assessed and confirmed using the Shapiro–Wilk test. The data were then analyzed using a 4 (all groups) by 2 (time: pre, post) analysis of variance (ANOVA) for repeated measures. Where the assumption of sphericity was violated, Greenhouse–Geisser correction was used to interpret the results.

If group × time interactions turned out to be significant, a simple contrast analysis was used. Additionally, effect sizes (ES) were determined from ANOVA output by converting partial eta-squared to Cohen’s *d*. Moreover, within-group ES were computed using the following equation: ES = (mean post-mean pre)/SD.

In accordance with Hopkins (2002), ES were considered to be either “trivial” (<0.2), “small” (>0.2–0.6), “moderate” (>0.6–1.2), “large” (>1.2–2), or “very large” (>2). Additionally, for single one leg hop tests measured just after the 6-week training program, comparisons between groups were performed with one-way ANOVA, and *post hoc* comparisons were calculated using Tukey-HSD test. Test–retest reliability of the variables was assessed using Cronbach’s model of ICCs and SEMs according to the method of Hopkins (2005).

For the varying groups, i.e., combined, plyometric, eccentric, and control, contrast analyses (Haans, 2008; Hervé and Williams, 2010) will also be carried out to specifically test the following hypotheses: (H1) COMB will yield greater improvements in the outcome measures than all other groups (PLYO, ECC, and CON), (H2) COMB will yield greater improvements in the outcome measures than PLYO and ECC, and (H3) COMB will yield greater improvements in the outcome measures than CON. Accordingly, three contrasts will be computed. Firstly, we will compare the COMB condition vs. PLYO, ECC, and CON conditions (coded as −0.75, 0.25, 0.25, and 0.25, respectively). The second will compare the COMB condition vs. PLYO and ECC (coded as −0.667, 0.333, and 0.333, respectively). Finally, the third contrast will compare the COMB condition vs. the CON condition (coded as −0.5 and 0.5, respectively). This approach yields a comparison of one (or more) condition(s) vs. the grand mean of the specified contrasts. Indeed, *post hoc* analysis, while useful, does not yield sufficient insight into multiple levels or detailing patterns in response, whereas contrast analysis allows researchers to test theory-driven expectations directly against empirically derived group or cell means (Rosnow and Rosenthal, 1995, 1996).

The level of significance was set at *p* < 0.05. The statistical analysis was carried out using IBM SPSS (version 25) and R (R Core Team, 2018) using the *Car: Anova* package (Fox Weisberg, 2018).

## Results

All 40 athletes from the experimental groups completed the study according to the study design and methodology. Participants attended all training sessions and none reported any training- or test-related injury.

Table 3 displays the intra session test–retest reliability analyses for all the tests conducted. Intra-class correlation coefficients showed good reliability for all tests. ICC values ranged from 0.85 to 0.97, with a standard error of measurement (SEM) from 0.02 to 7.45 and a coefficient of variation of <5%. Furthermore, a paired *t*-test showed no significant differences between the scores recorded during the two trials for all measured variables.

**TABLE 3:** Test-retest reliability of the applied tests.

	**Limb**	**ICC_3.1_ (95% CI)**	**SEM**	**CV (%)**
**Y-BAL (cm)**	Operated limb	0.85 (0.72–0.92)	1.17	4.05
	Non-operated limb	0.87 (0.75–0.93)	0.58	1.74
**One leg hop with hand (cm)**	Operated limb	0.89 (0.79–0.94)	1.79	4.85
	Non-operated limb	0.91 (0.83–0.95)	2.66	4.98
**One leg hop without hand (cm)**	Operated limb	0.95 (0.91–0.97)	1.29	4.82
	Non-operated limb	0.95 (0.90–0.97)	1.64	3.10
**Triple jumps (cm)**	Operated limb	0.92 (0.85–0.95)	7.45	4.99
	Non-operated limb	0.94 (0.88–0.97)	6.30	4.09
**Triple jumps cross (cm)**	Operated limb	0.91 (0.84–0.95)	7.37	4.92
	Non-operated limb	0.92 (0.86–0.96)	7.39	4.76
**6 m for the time (s)**	Operated limb	0.97 (0.95–0.98)	0.03	4.44
	Non-operated limb	0.96 (0.92–0.98)	0.02	3.23

*ICC, interclass correlation coefficient; SEM, standard error of measurements; CV (%), coefficient of variation percent, Y-BAL, dynamic stability.*

No significant between-group pre-training differences were found for any of the analyzed parameters (Table 4). Main effects of time across training were observed for all dependent variables (post-test > pre-test, *p* < 0.001).

**TABLE 4:** Comparison of dynamic stability, Lysholm Knee Scale, and return to sport index between experimental groups (ECC, PLYO, and COMB) and control group (CON) before and after the 6-week training programs.

		**Pre-intervention**	**Post-intervention**	**Delta%**	**Cohen’s *d* (pre vs. post)**	**ANOVA *p*-value (Cohen’s d)**		
						**Time**	**Group**	**Group × Time**
**Y-BAL (%)**	**CON**	71.80 ± 3.05	80.90 ± 2.85**	12.7	2.99	0.001 (3.96)	0.001 (2.29)	0.001 (1.73)
	**ECC**	71.10 ± 2.69	80.90 ± 2.85**	13.8	3.65			
	**PLYO**	75.20 ± 2.34	99.60 ± 3.69**	32.4^b,c^	5.62			
	**COMB**	74.70 ± 4.83	98.70 ± 1.57**	32.1^b,c^	4.97			
**LKS (AU)**	**CON**	61.60 ± 4.40	86.30 ± 4.69**	40.8	5.61	0.001 (5.17)	0.002 (0.60)	0.001 (0.76)
	**ECC**	60.30 ± 5.08	89.00 ± 4.08**	48.4^c^	5.65			
	**PLYO**	59.90 ± 5.82	89.90 ± 3.84**	50.9 ^c^	5.15			
	**COMB**	59.40 ± 4.93	96.00 ± 2.36**	62.6^a,b,c^	7.43			
**RSI (%)**	**CON**	50.13 ± 3.56	69.90 ± 3.06**	39.5	5.56	0.001 (4.89)	0.001 (2.20)	0.001 (1.39)
	**ECC**	49.32 ± 2.69	74.33 ± 3.78**	50.7	9.29			
	**PLYO**	51.80 ± 5.41	82.67 ± 4.28**	59.6	5.71			
	**COMB**	50.92 ± 3.35	92.80 ± 3.49**	82.2^a,b,c^	12.52			

**Significant difference from pre to post (*p* < 0.01).

*^*a*^Significant difference from PLYO (*p* < 0.001).*

*^*b*^Significant difference from ECC (*p* < 0.001).*

*^c^Significant difference from CON (*p* < 0.001). AU, arbitrary units; CON, traditional rehabilitation training; ECC, eccentric training; PLYO, plyometric training; COMB, combination of eccentric and plyometric training, Y-BAL, dynamic stability, LKS, Lysholm Knee Scale, RSI, return to sport index.*

Significant group-by-time interactions were found for Y-BAL (*p* < 0.001, effect size = 1.73), LKS (*p* < 0.001, effect size = 0.76), and RSI (*p* < 0.001, effect size = 1.39) (Table 4).

### *Post hoc* Analyses

For *post hoc* comparisons, both the COMB and PLYO groups made similar improvements of Y-BAL (+32.1%, ES = 4.97 and + 32.4%, ES = 5.62, respectively) more (*p* < 0.001) than ECC (+13.8%, ES = 3.65) and CON (+12.7%, ES = 2.99) groups (Table 4).

The COMB group showed greater improvement (*p* < 0.001) than the PLYO, ECC, and CON groups of LKS [COMB (+ 62.6%); PLYO (+ 50.9%); ECC (+ 48.4%); CON (+ 40.8%)] and RSI [COMB (+ 82.2%); PLYO (+ 59.6%); ECC (+ 50.7%); CON (+ 39.5%)] with the largest effect sizes for all groups (Table 4).

For jumping tests, the COMB group showed better performances in LSI than PLYO, ECC, and CON groups (*p* < 0.001) (Table 5). In addition, the PLYO group showed the best (*p* < 0.001) LSI compared to the ECC and CON groups. Finally, the ECC group is distinguished from the CON group (*p* < 0.001) (Table 5).

**TABLE 5:** Between group differences for the leg symmetry index during the single leg horizontally hop measured at post-intervention.

	**CON**	**ECC**	**PLYO**	**COMB**	**ANOVA *p*-value**
**One leg hop with hand (%)**	69.50 ± 2.92	84.60 ± 1.84^c^	91.50 ± 0.85^b,c^	99.60 ± 1.35^a,b,c^	0.001
**One leg hop without hand (%)**	68.20 ± 3.39	82.30 ± 1.83^c^	91.30 ± 1.34^b,c^	98.50 ± 0.53^a,b,c^	0.001
**Triple jumps (%)**	68.30 ± 3.77	83.30 ± 1.89^c^	91.30 ± 0.95^b,c^	98.90 ± 0.74^a,b,c^	0.001
**Triple jumps cross (%)**	64.80 ± 3.33	80.30 ± 2.00^c^	90.10 ± 1.20^b,c^	98.50 ± 0.71^a,b,c^	0.001
**6-meters for the time (%)**	68.10 ± 3.45	84.70 ± 2.67^c^	91.00 ± 0.67^b,c^	100.00 ± 1.41^a,b,c^	0.001

*^*a*^Significant difference from PLYO (*p* < 0.001).*

*^*b*^Significant difference from ECC (*p* < 0.001).*

*^*c*^Significant difference from CON (*p* < 0.001). CON, traditional rehabilitation training; ECC, eccentric training; PLYO, plyometric training; COMB, combination of eccentric and plyometric training.*

### Contrast Analyses

Specific to contrast analysis, all specified hypotheses were tested and are detailed in Table 5. For H3 (COMB vs. CON), we found that COMB yielded significantly greater improvements in Y-BAL, LKS, and RSI (all *p* < 0.0001), in addition to significantly better performances in LSI (all *p* < 0.0001) (Table 6).

**TABLE 6:** Contrast analyses of conditions for all variables.

		**Y-BAL**		**LKS**		**RSI**		**LSI**									
					
								**One leg hop with hand**		**One leg hop without hand**		**Triple jumps**		**Triple jumps cross**		**6 m time**	
	
		***P***	**Mean Diff**	***P***	**Mean Diff**	***P***	**Mean Diff**	***P***	**Mean Diff**	***P***	**Mean Diff**	***P***	**Mean Diff**	***P***	**Mean Diff**	***P***	**Mean Diff**
COMB	ALL	0.0001	9.56	0.0001	8.8	0.0001	16.7	0.0001	17.73	0.0001	17.9	0.0001	17.93	0.0001	20.1	0.0001	18.73
COMB	PLYO + ECC	0.0004	6.9	0.001	7.25	0.0001	13.9	0.0001	11.5	0.0001	11.7	0.0001	11.6	0.0001	13.3	0.0001	12.2
COMB	CON	0.0001	14.9	0.0001	11.9	0.0001	22	0.0001	30.1	0.0001	30.3	0.0001	30.6	0.0001	33.7	0.0001	31.9

*All differences are manifest from the grand means of the specified contrast(s). CON, traditional rehabilitation training; ECC, eccentric training; PLYO, plyometric training; COMB, combination of eccentric and plyometric training, Y-BAL, dynamic stability, LKS, Lysholm Knee Scale, RSI, return to sport index, LSI, leg symmetry index for the single leg hop test for distance.*

For H2 (COMB vs. PLYO and ECC) we found that COMB yielded significantly greater improvements in Y-BAL, LKS, and RSI (*p* < 0.001, *p* < 0.0001, *p* = 0.0004, and *p* < 0.0001, respectively), in addition to significantly better performances in LSI (all *p* < 0.0001) (Table 6).

Finally, for H1 (COMB vs. CON, ECC, and PLYO), we found that COMB yielded significantly greater improvements in Y-BAL, LKS, and RSI (all *p* < 0.0001), in addition to significantly better performances in LSI (all *p* < 0.0001) (Table 6).

## Discussion

The present study aimed to assess the effect of 6 weeks of eccentric training, plyometric training, or a combination of these two modalities (eccentric/plyometric), on measures of stability and functional performance in the post-ACL-surgical rehabilitation period of elite female athletes.

The most important findings were that the combination of eccentric and plyometric (COMB) exercises was more effective in improving Y-BAL, LKS, RSI, and LSI compared with ECC, PLYO, and CON programs.

In accordance with the concept of training specificity (Behm and Sale, 1993) and prior reported literature, it might be expected that a program that included combined PLYO and ECC activities would improve outcome measures to a greater extent than the other training types would alone. In support of this, a number of studies have demonstrated improved performance-related test scores with COMB programs after ACL reconstruction (Mandelbaum et al., 2005; Kinikli et al., 2014; Friedmann-Bette et al., 2018). Furthermore, we found an improvement in muscle functional performance, with the largest significant effects observed in the COMB group. These findings support the adaptive potential of muscle morphology in athletes after ACL reconstruction. Morphological changes following strength training can include an increase in muscle fiber size and changes in aspects of fiber-type composition and connective tissue, in addition to structural changes in the muscle itself (Friedmann-Bette et al., 2018).

In this context, Friedmann-Bette et al. (2018) demonstrated that strength training with an eccentric overload leads to significantly greater muscle hypertrophy than conventional strength training. The authors exposed recreational athletes to 12 weeks of strength training after which they observed that the eccentric group achieved greater increases in fiber cross-sectional areas of all muscle fiber types, displaying a significant correlation with increases in muscle strength. The addition of an eccentric overload in this case corroborates our findings with the ECC group achieving functional performance increase, an adaptation that was enhanced in the COMB group that added plyometric exercise to the rehabilitation protocol.

The combination of these results suggests that different mechanisms may play a role in contributing to a comprehensive rehabilitative with increases in muscle mass and strength being seemingly bolstered by the additive effect of plyometric training, which taxes the neuromuscular system and associated stretch shortening-cycle capabilities (Komi, 2003). Substandard movement mechanics, such as excessive lateral displacement of the trunk or asymmetric limb loading, play an influential role in creating valgus forces at the knee joint, increasing the risk of injury (Nessler et al., 2017). In females, this risk seems to be elevated given a higher level of knee laxity and a lower propensity to utilize the hamstrings to resist forces exerted on the ACL (Huston and Wojtys, 1996). Furthermore, female athletes can, supposedly, benefit from combined strength and plyometric training, due to the low baseline levels of strength and power compared to their male counterparts.

These training programs for women can improve many performance measures such as speed, strength, and power (Kraemer et al., 2003; Myer et al., 2005). Combined strength and plyometric training is a wide-ranging term and can be characterized as facilitating sudden movement-based responses during rapid changes in the positioning of a joint (Myer et al., 2005). Furthermore, the term encapsulates plyometric exercise that can be highly beneficial for postural control and dynamic stability when it induces unconscious efferent responses to afferent stimuli. Accordingly, our results revealed that the injured limb displayed greater dynamic stability for maximal anterior reach, maximal posterolateral reach, and composite scores after COMB than after PLYO or ECC training, respectively, which were both still highly effective in this regard. However, it seems that the addition of strength and plyometric exercise to the traditional protocol was additive in this case, underlining the efficacy of the COMB protocol.

An athlete’s balance can be improved with a strength training program that enhances the ability and speed of postural muscles to return to a more stable position following perturbation (Anderson and Behm, 2005). Plyometric training comprises of a dynamic form of resistance training involving a rapid stretch-shortening cycle that can involve both vertical and horizontal displacement of the center of gravity, improving an individual’s anticipatory postural adjustment. Anticipatory postural adjustment challenges result in proactive or feed-forward adjustments that stiffen the involved musculature, prior to landing (Sparkes and Behm, 2010).

Furthermore, the sensitivity of afferent feedback pathways may be improved with balance and motor skill training, resulting in a faster onset of stability (Anderson and Behm, 2005). The dynamic nature of the COMB exercises could enhance motor control and leg stiffness, which, in turn, could improve postural control or equilibrium (Anderson and Behm, 2005). The International Knee Documentation Committee Subjective Knee Form Documented Data indicates the effectiveness of plyometric exercises in improving functional knee assessment (Irrgang et al., 2006) with post-intervention outcomes approaching those of patients who returned to sport 1 year after surgery (Lentz et al., 2015).

Furthermore, the execution of strength and plyometric training may be an important determinant of functional knee assessment as an injured individual age. For instance, plyometric training has been associated with positive outcomes relating to bone mass (Xu et al., 2016). Indeed, as individuals advance in age, the advantage of combined resistance and plyometric training over plyometric training only is emphasized, as in the current study (Xu et al., 2016). This seemingly facilitates a more comprehensive adaptation by way of differing adaptive responses to the respective exercise’s protocols. Along these lines, it was previously demonstrated that plyometric activity enhanced eccentric force production in the lower body to a greater degree than traditional resistance training, which predominantly favored concentric adaptations and subsequently may have a positive effect on functional knee assessment in general (Wilson et al., 1996). This is accentuated in females given the aforementioned imbalance in hamstring activation, in addition to greater knee extension angles, during landing tasks (Quatman et al., 2010).

This can lead to the development of an efficient stretch-shortening cycle, particularly important for deceleration movements (Moran et al., 2018) such as landing and turning, which can generate forces that the ACL might be unable to accommodate (Shelbourne et al., 1991).

Furthermore, the present study demonstrated that the COMB group outperformed the PLYO, ECC, and CON groups on RSI scores. The probable explanation was that the protocol of the COMB group imposed a more comprehensive stress of greater magnitude and improved psychological status by way of increased confidence levels for the athlete. This assertion is supported by Ardern et al. (2011) who highlight the discrepancy between the function required to RTS and that required to resume full physical capability. It is plausible that the difference between these two states is representative of the gap between confidence to play, and a lack thereof, in the post-operative period with fear of re-injury an influential factor in an individual’s psychological state (Ardern et al., 2011).

It is possible that the comprehensive nature of a COMB training stimulus is beneficial in this regard as it may provide athletes with the confidence they require to return to competitive play. The relatively low rate of return to competitive sport, despite high rates of successful surgical outcomes, supports the use of psychological interventions as being part of a multidimensional approach to ACL rehabilitation, alongside physical activity programs that closely reflect the demands of the athlete’s sport.

This study is not without limitations. First, the sample size is small in each group and limited the statistical power and the generalizability of the findings. Furthermore, the study participants were highly homogeneous (i.e., female athlete’s post-ACL returning to sport), which limits the generalizability of its findings. Second, subjects have a narrow age range. Moreover, a lack of nutritional control and of activities outside of the training program most certainly could be considered as factors to potentially impact our outcomes. Third, systematic bias due to learning effects within our participants cannot be completely ruled out. However, they were used in most of the testing protocols performed (i.e., balance and jumping) and had also been part of a familiarization session 1 week before the start of the study. As such, although learning effects may be one possible explanation for the present results, they should be negligible. Finally, another limitation is the simplicity of the applied fitness tests (e.g., simple hopping, jumping, and Y-balance tests), which did not extend the current mechanistic knowledge. In fact, the present variable used in the current study may be too broad and does not allow for clear speculation of the possible physiological mechanisms explaining the positive effect of combining eccentric and plyometric training for performance enhancement after an ACL-surgical rehabilitation period of elite female athletes. Therefore, future studies are advised to include research approaches to elucidate the underlying neural changes.

## Conclusion

Based on our results, it is apparent that, despite all of the training methods inducing improvement outcomes to various extents, combined (eccentric/plyometric) training was the most effective protocol to stimulate positive changes in Y-BAL, LKS, RSI, and LSI index. On this basis, combined protocol represents the most efficacious training program as it exerts positive effects on both stability and functional performance in the post-ACL-surgical rehabilitation period of elite female athletes.

## Data Availability Statement

The raw data supporting the conclusions of this article will be made available by the authors, without undue reservation.

## Ethics Statement

This study was conducted according to the Declaration of Helsinki and the protocol was fully approved by the Ethics Committee of the National Centre of Medicine and Science of Sports of Tunis (CNMSS-LR09SEP01) before the commencement of the assessments. The patients/participants provided their written informed consent to participate in this study.

## Author Contributions

SK, RH, AH, FS, HZ, and OO conceived the manuscript ideas and design. SK, UG, ACH, AH, MC, CC, and OO drafted, revised, and edited the manuscript. SK, MC, FS, CC, SC, ACH, UG, HZ, and OO assisted in revising and editing the manuscript. All authors have read and approved the final version of the manuscript, and agreed with the order of the presentation of the authors.

## Conflict of Interest

The authors declare that the research was conducted in the absence of any commercial or financial relationships that could be construed as a potential conflict of interest.
